# The Expression and Potential Role of MicroRNAs in Oral Lichen Planus

**DOI:** 10.1111/jop.70122

**Published:** 2026-02-03

**Authors:** Mousa Ali Heba, Resteu Anastasia, Werner Andreas, Carrozzo Marco

**Affiliations:** ^1^ Biosciences Institute Newcastle University Newcastle upon Tyne UK; ^2^ Oral Medicine Department, School of Dental Sciences Newcastle University Newcastle upon Tyne UK; ^3^ Translational and Clinical Research Institute, Newcastle University Newcastle upon Tyne UK; ^4^ NIHR Biomedical Research Centre Newcastle Univerity Newcastle upon Tyne UK

**Keywords:** malignant transformation, microRNAs, oral lichen planus, pathogenesis

## Abstract

**Background:**

Oral lichen planus (OLP) is a chronic T‐cell‐mediated immune disease of unknown aetiology. MicroRNA (miRNAs) are short non‐coding RNAs capable of regulating mRNA and may have roles in T‐cell‐related diseases. The aim of this study was to investigate the profile of miRNAs in OLP patients and its interaction with potential target genes.

**Methods:**

Total RNA was extracted from fresh frozen biopsies from 24 patients with OLP and 8 control patients. The NanoString Counter Analysis System was used to analyse total RNA samples for differential miRNA expression. NanoString expression was confirmed by RT‐qPCR analysis. Genes potentially targeted by upregulated and downregulated miRNAs were identified, and RT‐qPCR was employed to investigate the expression of target genes in OLP and controls. Probability values < 0.05 were considered statistically significant.

**Results:**

NanoString analysis showed that eight miRNAs, miR‐155, miR‐146a, miR‐3195, miR‐342, miR‐4516, miR‐21, miR‐29a and miR‐193 were upregulated in OLP tissues. Contrarily, the other eight miRNAs, miR‐221, miR‐200b, miR‐149, miR‐205, miR‐27b, miR‐95, miR‐127b, miR‐95, miR‐206 were downregulated in OLP tissues. NanoString findings have been confirmed by RT‐qPCR results for four upregulated miRNAs, miR‐155, miR‐146a, miR‐29a and miR‐342, and one downregulated miRNA, namely miR‐205. The expression of two target genes, namely MYC for miR‐29a and interleukin‐24 (IL‐24) for miR‐205, was found to negatively correlate with the respective miRNA. This suggests that MYC and IL‐24 could be regulated by the above miRNAs in OLP.

**Conclusions:**

The work presented in this study suggests that miRNAs could be involved in both the immunopathogenesis and malignant transformation of OLP.

## 1 Introduction

Oral lichen planus (OLP) is a chronic oral mucosal disease frequently of unknown cause [1]. OLP lesions rarely undergo spontaneous remission; they can cause significant pain and morbidity, and evidence suggests that they can undergo malignant transformation [1]. OLP likely represents a T‐cell‐mediated immunological response to an unknown antigenic change in the oral mucosa in predisposed patients [2]. An early event in OLP pathogenesis is the increased production of Th1 cytokines, particularly interferon‐gamma (IFN‐γ) and tumour necrosis factor‐alpha (TNF‐α), that is thought to be genetically driven [3].

MicroRNAs (miRNAs) are 20–22 nucleotide‐long non‐coding RNA molecules first discovered in 1993 [4]. miRNAs are involved in the control of both innate and adaptive immunity [5]. Recent studies have suggested possible roles for miRNA regulation in several T‐cell‐related autoimmune diseases, including systemic lupus erythematosus (SLE), rheumatoid arthritis (RA), multiple sclerosis, inflammatory bowel disease, Sjogren's syndrome and Type I diabetes mellitus [6]. Persistent and excessive cytokine production is a hallmark of autoimmune diseases, and altered miRNA levels observed in most autoimmune diseases likely impact the production of pathogenic cytokines [6]. As OLP is a T‐cell‐mediated, localised autoimmune disorder characterised by altered expression of cytokines [7, 8], miRNAs may be involved in the pathogenesis of OLP.

Previous studies have analysed miRNA expression in OLP [9, 10, 11, 12, 13, 14, 15, 16, 17]. However, only sporadically, other techniques jointly confirmed microarray/RNA‐sequencing findings.

The aims of this study were to investigate with a genome‐wide analysis the miRNA profile in OLP patients and controls and to confirm the findings using reverse transcription‐PCR (RT‐qPCR). Moreover, we have analysed potential targets of differentially expressed miRNA with RT‐qPCR.

## 2 Materials and Methods

Oral mucosa biopsy specimens were collected from patients clinically and pathologically diagnosed with OLP according to the 2003 World Health Organisation diagnostic criteria [18] at the Department of Oral Medicine, Newcastle Dental Hospital, between March 2014 and February 2018.

Other oral lichenoid disorders, diagnosed according to Carrozzo et al. [1], were excluded. As a control group, patients matched for age and gender with OLP and with other oral disorders causing white patches and diagnosed according to clinical–pathological standard criteria were enrolled. All samples were suspended in RNA‐later and immediately transferred into liquid nitrogen until RNA extraction. All experimental procedures were approved by the NRES Committee Northeast—Newcastle & North Tyneside (13/NE/0368). The enrolled patients gave their informed written consent.

### 2.1 RNA Extraction

Total RNA was extracted from fresh frozen biopsies using an RNeasy Mini Kit (Qiagen, Manchester, UK) according to the manufacturer's protocol. The quality and the quantity of RNA were determined using an Agilent 2100 bioanalyzer (Agilent, Santa Clara, USA). The results were confirmed using Nanodrop (Thermo Scientific, Paisley, UK) and gel electrophoresis 2% agarose (tris‐acetate–EDTA buffer).

### 2.2 NanoString Analysis

The multiplexed NanoString nCounter miRNA expression assay (NanoString Technologies, Seattle, WA, USA) was used to profile more than 800 human miRNAs. Total RNA samples with a concentration of 30 ng/μL were used. The experimental quantification was performed by the NanoString facility at Newcastle University Medical School, and NanoString protocols were strictly followed. Differential miRNA expression was quantified using the NanoString nCounter FLEX platform and the nSolver software version 4.0 to normalise the data, using the geometric means of NanoString's internal positive and negative controls. miRNAs with low expression were filtered out with the genefilter R package version 1.84.0. Only miRNAs with at least 30 counts in ≥ 50% of samples were retained for downstream analysis. Data filtering and analysis were undertaken in R (version 4.3.3) using NanoStringDiff (version 1.10.0) to identify differentially expressed miRNAs. Log2‐fold changes were estimated using a negative binomial‐based generalised linear model.

### 2.3 RT‐qPCR

To mitigate the fact that miRNAs are short and without a poly A tail, the miScript II RT Kit (Qiagen) was used and the supplier's protocol was strictly followed. The mature miRNAs were polyadenylated by poly(A) polymerase and then reverse transcribed into cDNA using an oligo‐dT primer with a unique sequence tag. An miRNA‐specific forward primer and a primer complementary to the adaptor were used for qPCR. Two microlitres of RNA template (OKF6 cell line RNA) in a final volume of 10 μL was used. This mixture was incubated at 37°C for 60 min, followed by a heat denaturation step. Samples were diluted (1/3 and 1/5) and stored at −20°C.

For mRNA quantification, the Omniscript RT‐PCR kit (Qiagen) was used for cDNA synthesis.

Ten‐microlitre reactions containing 2 μL of RNA template was assembled and processed according to the supplier's instructions.

Three specific primer pairs synthesised by Integrated DNA Technologies (IDT) per gene were tested, and the most reliable pair was used for mRNA quantification by qPCR. The SyBR Green Master Mix (Roche) and a QuntaStuido 3 Real‐Time PCR machine (Thermo Fisher Scientific) were used to detect the amount of DNA template in the samples.

### 2.4 Target Gene Selection

Potential target genes for selected differentially expressed miRNA were chosen using the following web tools:

1. Target Scan human (www.targetscan.org)
2. DIANA (www.diana.imis.athenainnovation.gr/DianaTools)
3. Exiqon (www.exiqon.com/miRSearch)
4. miRTarBase (www.mirtarbase.mbc.nctu.edu.tw)

For each miRNA, the most predicted targets identified by the four predicting websites were compiled. The predictions were then assessed for a potential involvement in OLP pathogenesis with the aim of minimising the number of target genes.

### 2.5 Statistical Analysis

Statistical analysis for the NanoString differential expression was determined by *t*‐test. Hierarchical clustering was performed to visualise the relationships between groups using Ward's method (ward.D2) with squared Euclidean distances within the hclust function from the stats package. Statistical analysis of qPCR results was performed by Mann–Whitney *U* test. *p* ≤ 0.05 were considered statistically significant. The relationship between miRNA and target mRNA was determined using the Spearman correlation coefficient test. Correlation was categorised as follows: *r* < 0.25, no relationship; *r* between 0.25 and 0.5, weak relationship; *r* between 0.5 and 0.75, moderate relationship; *r* > 0.75, strong relationship.

## 3 Results

### 3.1 Patients and Controls

Oral biopsy samples were collected from 24 patients with OLP (12 females and 12 males with an average age of 57.37). A total of 12 patients had reticular lesions of OLP: 6 reticular/atrophic, 5 reticular/patch‐like and 1 reticular/erosive.

In addition, eight samples (five females and three males with an average age of 56.4) represented the control group. Four control patients had a final diagnosis of leucoplakia without dysplasia, two were affected by chronic hyperplastic candidiasis (candida leucoplakia), one had leucoplakia with mild dysplasia and the last one was diagnosed with oral hairy leucoplakia (Table S1).

### 3.2 NanoString Analysis

**FIGURE 1:**
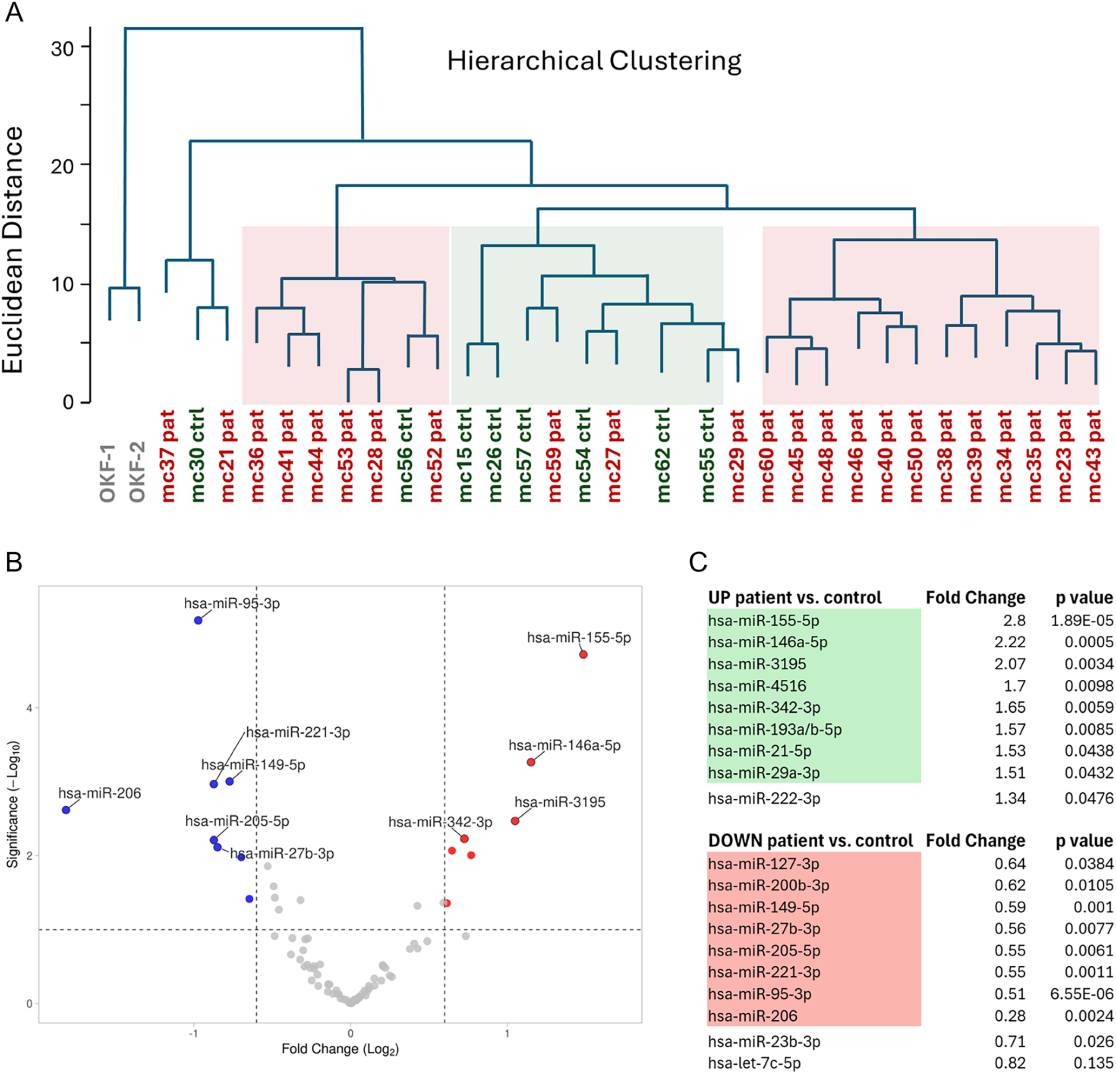
Differential expression of miRNA in OLP patients versus controls. (A) Cluster dendrogram of individual OLP patients and control subjects related to miRNA expression. (B) Volcano plot of expressed miRNAs in OLP patients versus controls. A 1.5‐time expression change and a *p* value of < 0.05 were the criteria for differential expression. (C) List of differentially expressed miRNAs. Green shade indicates miRNA overexpressed in OLP, red shade indicates repression of the miRNA in OLP patients as compared to controls. The three miRNAs miR‐222, miR‐23b and let‐7c were included in further analysis as references for unchanged (let‐7c) and borderline calls (miR‐222, miR‐23b).

The expression profile of 799 cellular miRNAs was assessed in OLP (*n* = 24) and controls (*n* = 8) as well as OKF6 cells (*n* = 2). A hierarchical cluster analysis was produced, shown in Figure 1A. The majority of the OLP patient samples are clustered in two groups (shaded red). In contrast, most control samples are segregated in the green cluster (7/9). The control, including the OKF cell lines, showing a clearly separated cluster (OKF‐1 and OKF‐2), validates the distinct miRNA signature in OLP.

Of the total miRNAs tested, 82 were expressed above background. Eight miRNAs were significantly upregulated in OLP compared to controls (miR‐155‐5p, miR‐146a‐5p, miR‐3195, miR‐4516, miR‐342‐3p, miR‐193a/b‐5p, miR‐21‐5p and miR‐29a‐3p). In contrast, eight miRNAs exhibited lower expression in OLP patients (miR‐127‐3p, miR‐200b‐3p, miR‐149‐5p, miR‐27b‐3p, miR‐205‐5p, miR‐221‐3p, miR‐95‐3p, hsa‐miR‐206) (Figure 1B,C).

### 3.3 RT‐qPCR Analysis of miRNA Expression in OLP Patients and Controls

**FIGURE 2:**
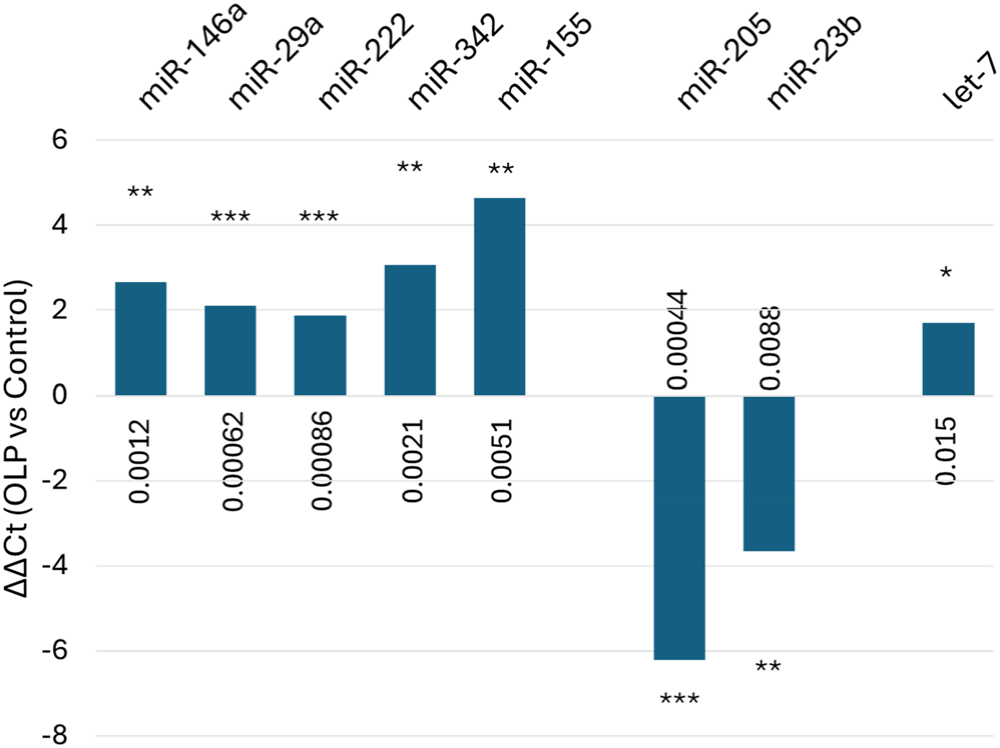
Expression analysis of selected miRNA in OLP versus control patients by RT‐qPCR. The comparison included 10 patient samples and 8 controls. The median of ΔΔ*C*_t_ values related to β‐actin is displayed, *p* values were determined by Mann–Whitney test, *p < 0.05, **p < 0.01, ***p < 0.001.

The expression results of selected miRNAs were confirmed using RT‐qPCR from samples of 10 OLP patients and 8 controls. Four upregulated miRNAs in OLP patients, miR‐146a, miR‐342, miR‐155 and miR‐29a, were confirmed by RT‐qPCR (*p* < 0.01) (Figure 2, Table S2). Likewise, the expression of downregulated miR‐205 and miR‐23b was significantly reduced between OLP and control groups (Figure 2). In contrast, let‐7 and miR‐222 expressions (which were not significantly altered between OLP and controls according to NanoString) were significantly increased in OLP patient samples compared to the control group (*p* = 0.015 and 0.00086, respectively) (Figure 2, Table S2).

### 3.4 RT‐qPCR Analysis of Potential miRNA Target Genes

**FIGURE 3:**
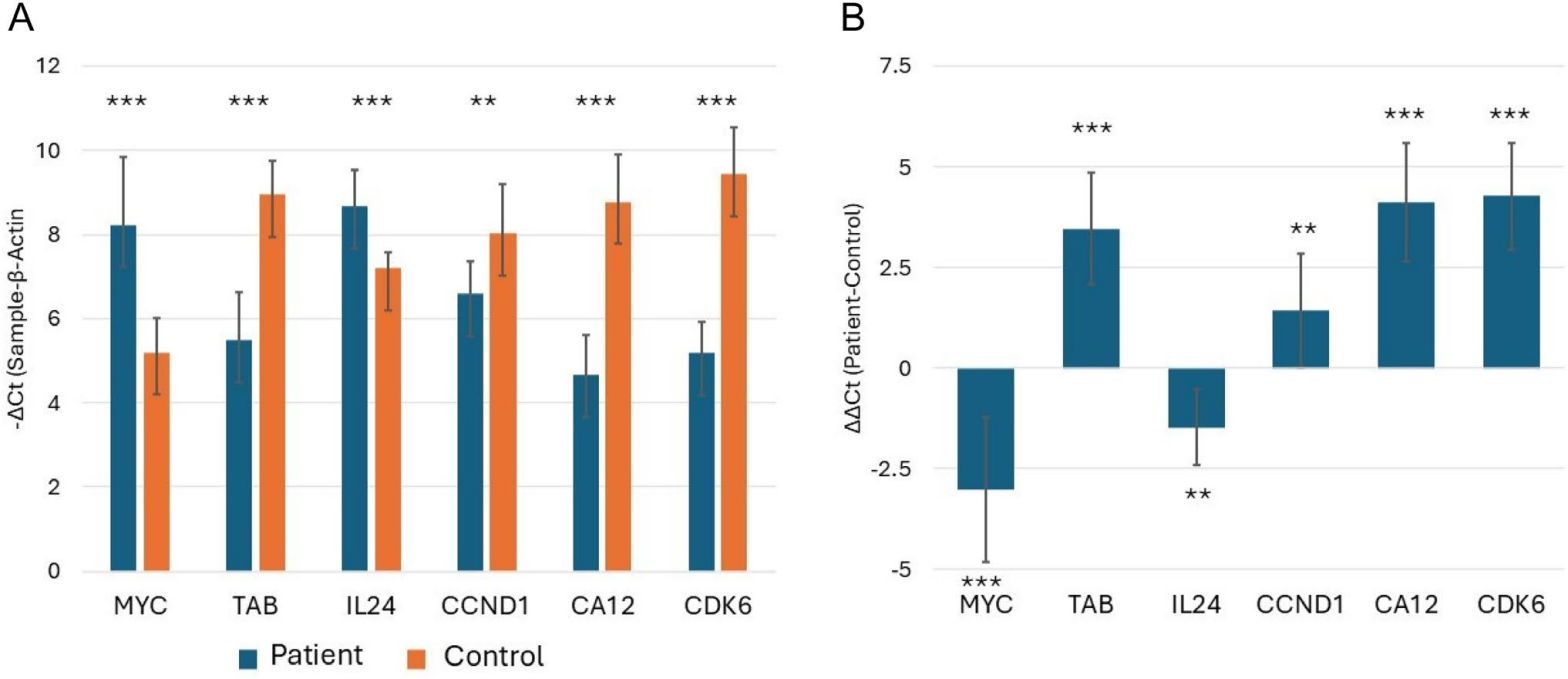
RT‐qPCR of potential miRNA target genes in patients with OLP and controls. The comparison included 10 patient samples and 8 controls. (A) The average of −Δ*C*_t_ values ± standard deviation related to β‐actin are shown. Blue bars patients, red bars controls. Of note, high bars represent low expression and vice versa. (B) ΔΔ*C*_t_ values, OLP patients versus controls. *p* values:**p < 0.01, ***p < 0.001.

We considered the first 100 target genes of each web tool and identified the ones called by all four predicting websites. The lists were further parsed to focus on potential target genes with an established role in OLP. The mRNA expression levels of *MYC*, *IL24*, *CCND1*, *TAB*, *CA12* and *CDK6* were assessed. The mRNA levels of MYC and IL24 were significantly decreased in OLP samples as compared to the control group (8.14‐fold, *p* < 0.001 and 2.77‐fold, *p* < 0.005, respectively, Figure 3, Table S2). The expression level of the target gene CCND1 was only slightly increased in OLP patients compared to controls (*p* < 0.01, Figure 3A, Table S2). For the target genes *CDK6*, *CA12* and *TAB2*, mRNA expression levels in OLP patients were significantly increased in comparison to the control group. The fold change between patients and controls and the values were 19.37 (*CDK6*), 17.46 (*CA12*) and 11.03 (*TAB2*), respectively (*p* < 0.001) (Figure 3B, Table S2).

### 3.5 miRNA–mRNA Interaction

**FIGURE 4:**
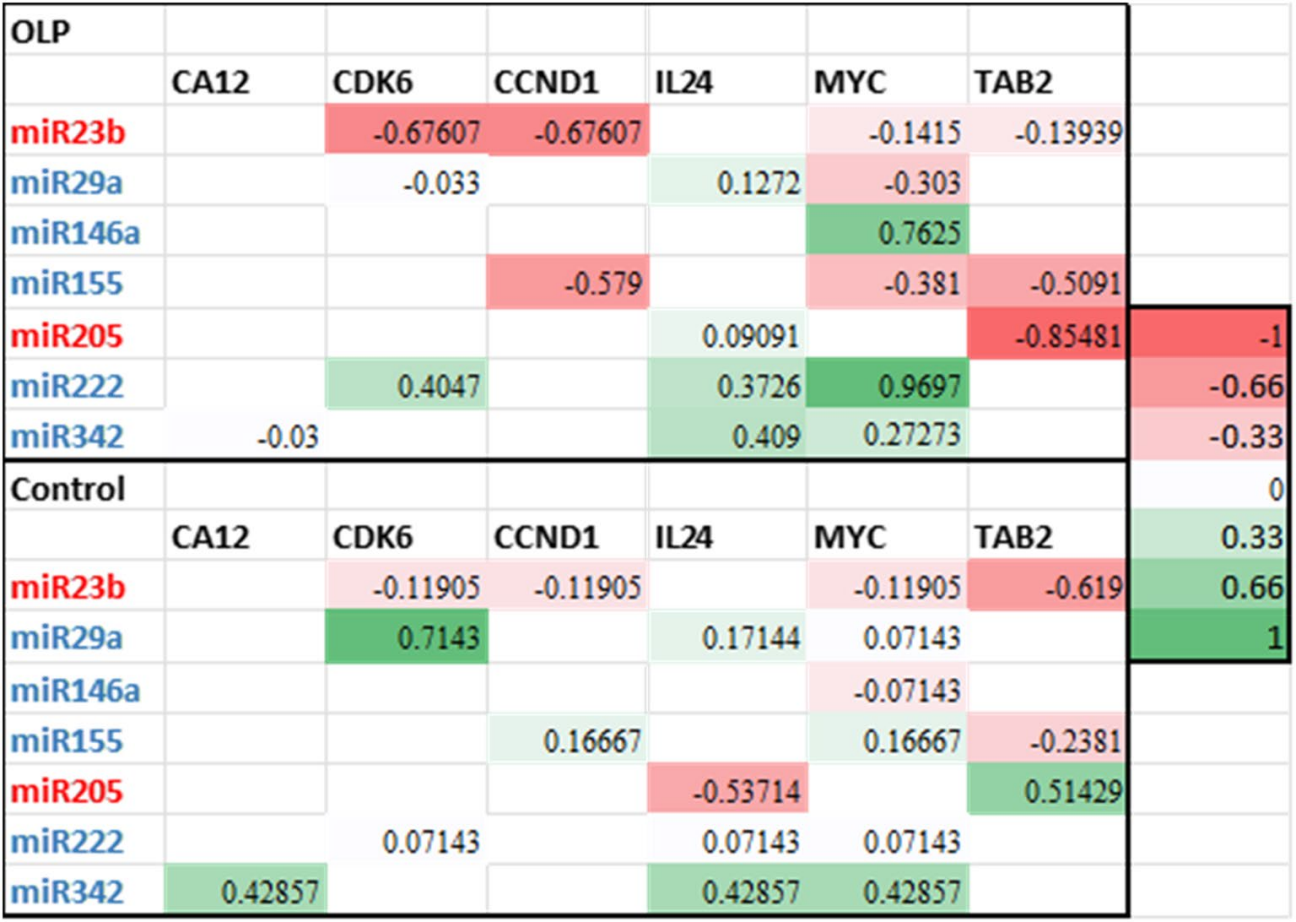
Spearman correlation between miRNAs and potential target genes. For a canonical miRNA–mRNA interaction a negative correlation would be expected, shown in red shapes. Positive correlation is shown in green. The numbers indicate the Spearman coefficient determined between expression levels of miRNA and mRNA determined by RT‐qPCR.

According to the assumption that high miRNA levels have a negative impact on the level of their target genes, a negative correlation would be expected between miRNA levels and the target gene in individual samples. Correlations were calculated for the two downregulated miRNAs (miR‐205 and miR‐23b) and the five upregulated miRNAs (miR‐155, miR‐146a, miR‐222, miR‐29a and miR‐342) with their six potential target genes (*MYC*, *CCND1*, *CDK6*, *TAB2*, *IL24* and *CA12*). In Figure 4, the result of the analysis is summarised. Accordingly, strong negative associations in patient samples could only be detected for miR‐205 and *TAB2*. Moderate negative correlation was found for miR‐23b/*CDK6* and miR‐23b/*CCND1* as well as miR‐155/*CCND1* and miR‐155/*TAB2*. A weak negative relation was seen for miR‐29a/MYC and miR‐155/MYC (Figure 4, Table S2). In control samples moderate correlation was detected for miR‐23b/*TAB2* and miR‐205/*IL24*.

**FIGURE 5:**
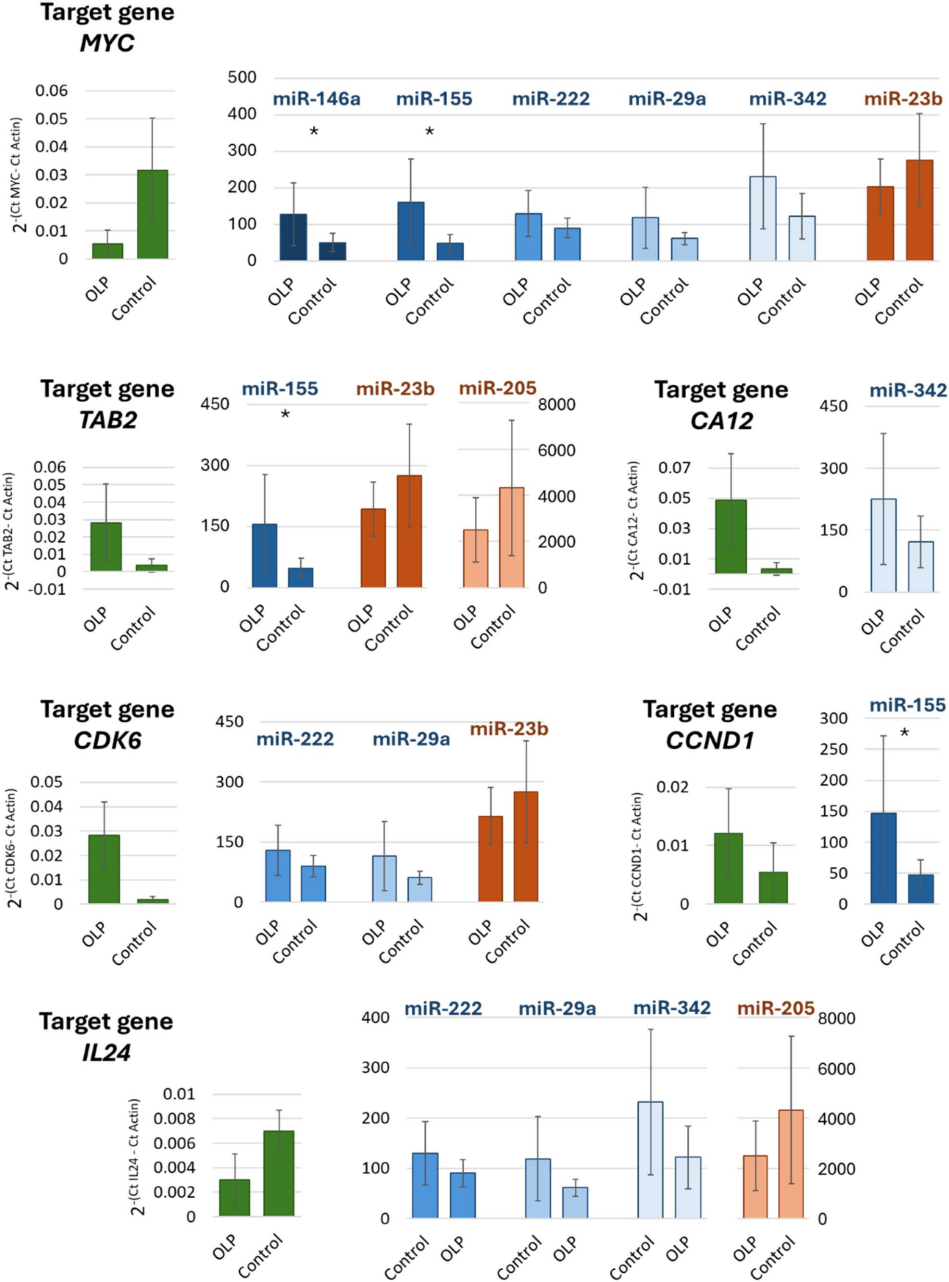
Quantitative analysis of miRNA and proposed target genes. The expression levels of the target genes were determined in 10 selected OLP patient samples and 8 controls. Average expression levels of the target genes and the standard deviation are depicted in green (all differences are significant, see Figure 4). The expression levels of the different miRNAs are taken from the NanoString results, blue bars indicate increased expression in OLP patients, terracotta means decreased levels. *p* values: *p < 0.05.

The level of expression for the miRNAs in each sample obtained from the NanoString analysis was compared to the expression levels of the related mRNAs in the same sample (Figure 5). An inverse relationship between miRNA and potential target mRNA could be established between miR‐146a and miR‐155 and *MYC* (Figure 5). Comparable slight inverse expression (though not statistically significant) was found for IL24 with miR‐222, miR‐29a and miR‐342, as well as for CDK6 with miR‐23b (Figure 5).

## 4 Discussion

This is the first study on miRNA expression in British patients with OLP. Our findings suggest that eight miRNAs were upregulated in OLP, namely miR‐155, miR‐146a, miR‐3195, miR‐342, miR‐4516, miR‐21, miR‐29a and miR‐193a, while eight other miRNAs, miR‐127, miR‐200b, miR‐149, miR‐27b, miR‐205, miR‐221, miR‐206 and miR‐95, were downregulated. One upregulated miRNA, miR‐4516 and two downregulated miRNAs, miR‐149 and miR‐95, were novel discoveries, whilst the remaining miRNAs were confirmation of previous findings [9, 10, 11, 12, 13, 14, 15, 16, 17]. Two others, miR‐3195 and miR‐193, were determined to be upregulated, but previous studies found them mainly downregulated in OLP [12]. Different from many previous microarray studies, our NanoString findings have been confirmed by qPCR results for the following four upregulated miRNAs: miR‐155, miR‐146a, miR‐29a and miR‐342, and one downregulated miRNA, namely miR‐205.

Other studies not only used different methods to investigate miRNA expression, but they also employed different sources of miRNAs, either peripheral blood or saliva [13, 15]. Whereas in typical autoimmune disorders such as SLEs or RA, peripheral blood mononuclear cells (PBMCs) may represent a reliable source of information to investigate immune pathogenesis, this is less straightforward in OLP as it is a more localised autoimmune disease [1]. Similarly, saliva can be a potentially helpful source of biomarkers for oral disorders, but managing confounding factors such as, for example, periodontal disease, may be challenging.

It is well‐known that miR‐155 is essential for immune system regulation, but the underlying mechanisms in OLP have not yet been fully elucidated. Hu et al. [16] ascertained that whilst miR‐155 negatively regulates suppressor of cytokine signalling 1 (SOCS1) in CD4^+^ T lymphocytes, it has a positive interaction with IFN‐γ, which may facilitate a T helper type 1 cell (Th1) polarisation in OLP.

Similarly to miR‐155, miR‐146a has been found to be mostly overexpressed in OLP [12, 14, 17].

The other two upregulated miRNAs, namely miR‐29a and miR‐342, and the one downregulated, miR‐205, whose expression has been confirmed by qPCR in our study, could be involved in malignant transformation of OLP more than its immunopathogenesis. miR‐29a plays indeed a pivotal role in cancer metastasis [19], whereas miR‐342 has been linked to melanoma prognosis [20]. Aberrant expression of miR‐205 is frequently found in human cancers, where it was reported to act either as tumour suppressor or an oncogene depending on the specific tumour context and target gene [21].

Among the target genes studied, MYC, which was one of the predicted target genes for the upregulated miR‐29a, was significantly downregulated in OLP. According to these results, miR‐29a could be directly regulating MYC in OLP. Of note, it has been reported that MYC status on some OLP lesions may predict a subgroup of patients with a higher risk of progression to oral squamous cell carcinoma (OSCC) [22]. MYC expression levels were also associated with the advanced stages of oral epithelial dysplasia and OSCC, wherein MYC and p53 are overexpressed in the early stages of oral carcinogenesis [23].

Target gene prediction identified IL‐24 as a potential target for the downregulated miR‐205. RT‐qPCR experiments revealed that in OLP patient samples, IL‐24 was upregulated as compared with the control.

IL‐24 was isolated in 1996 and then characterised as a member of the IL‐10 family of cytokines [24].

IL‐24 was found upregulated in psoriasis, atopic dermatitis and SLE [25, 26]. Current data suggest that IL‐24 may display both pro‐ and anti‐inflammatory properties [24]. Moreover, IL‐24 is also a well‐known tumour‐suppressor gene in a variety of cancers, including OSCC [27].

This study is apparently the first showing abnormal expression of IL‐24 in OLP. This cytokine could be involved in both immunopathogenesis and malignant transformation of the disease, and our findings suggest that IL‐24 expression could be regulated by miR‐205.

Although our study provided valuable insights into the role of miRNAs in OLP, it has some limitations, particularly regarding the relatively small sample size and the fact that our findings are largely based on differential expression. Furthermore, more specific studies are required for functional validation of our results.

## 5 Conclusions

We screened up to 800 miRNA genes and found eight upregulated and eight downregulated miRNAs in OLP tissues. We confirmed the results using RT‐qPCR for four upregulated (miR‐155, miR‐146a, miR‐29a and miR‐342) and one downregulated (miR‐205) miRNAs. Our results suggest that miRNAs could be involved in both the immunopathogenesis and malignant transformation of OLP, as shown by a variety of possible interactions with target genes, specifically MYC and IL‐24.

## Author Contributions

C.M. contributed to the study design, recruitment of the patients, target gene selection and writing and reviewing the manuscript. W.A. contributed to the study design, RT‐qPCR and NanoString, target gene selection, acquisition of data and writing and reviewing the manuscript. M.A.H. contributed to RT‐qPCR, NanoString, target gene selection and writing and reviewing the manuscript. R.A. contributed to NanoString, acquisition of data and statistical analysis and reviewing the manuscript.

## Funding

This work was supported by the Libyan Embassy.

## Conflicts of Interest

The authors declare no conflicts of interest.

## Supporting information


**Data S1:** Supporting Information.

**Table S1:** Demographic and diagnosis of cases (OLP) and controls.

**Table S2:** Average and standard deviation (SD) and *p* values of the figures.

## Data Availability

The data that support the findings of this study are available on request from the corresponding author. The data are not publicly available due to privacy or ethical restrictions.
