# Supplementary material for: The Expression and Potential Role of MicroRNAs in Oral Lichen Planus
Source: J Oral Pathol Med. 2026 Feb 3;55(6):642–9. doi: 10.1111/jop.70122 (PMC13333531; doi:10.1111/jop.70122)
Supplement: Supplementary file 1 — Data S1: Supporting Information. [file JOP-55-642-s003.docx]

**Supplementary material 1**

**Nano String analysis**

All probe sequences were specifically designed and manufactured by means of NanoString. A number of housekeeping genes are included in each code set to account for variations in RNA input amount and/or quality. Housekeeping genes were chosen from publicly accessible databases for their stability and measurable expression levels in the tissue types of interest. While one Code Set had four housekeeping genes, the others all had at least eight.

The nCounter miRNA expression assay was run on the nCounter Analysis system. The system consists of two instruments, the Prep Station used for post-hybridisation processing and the Digital Analyser used for data collection.

All experiments included twelve samples. The protocol started with diluting the miRNA Assay control and preparation of the annealing master mix followed by the ligation protocol as by the manufacturer’s instructions. After adding ligation clean-up enzyme to each reaction they were ready for purification according to the supplier’s protocol. At that point, the samples can be stored at -20°C for several weeks, before proceeding with the miRNA CodeSet Hybridisation protocol.

After hybridisation, excess probes were washed away using a two-step magnetic bead- based purification on the nCounter Prep Station. Magnetic beads derivative with short nucleic acid sequences that are complementary to the Capture Probe and the Reporter Probes were used sequentially. The hybridisation mixture containing target/probe complexes was then allowed to bind to magnetic beads complementary to sequences on the Capture Probe.

Wash steps were completed to remove excess Reporter Probes and non-target cellular transcripts. After washing, the Capture Probes and target/probe complexes were eluted off the beads and were hybridised to magnetic beads complementary to sequences on the Reporter Probe. An additional wash was performed to remove excess Capture Probes. Lastly, the purified target/probe complexes were eluted off the beads and immobilised on the cartridge for data collection.

Data Collection was carried out in the nCounter Digital Analyser. Digital images were processed and the barcode counts were arranged in a comma separated value (CSV) format.

The NanoString n Counter Analysis System (NanoString Technologies®, Inc.), was utilised to analyse total RNA samples with a concentration of 30µg/µl. The quantification of the miRNAs was performed in the NanoString facility at Newcastle University Medical School.

**miRNA sample preparation**

Total RNA was used as starting material in the nCounter miRNA expression assay. NanoString protocols recommend using 100 ng of total RNA.

Total RNA samples were prepared using RNAse-free water to dilute the total RNA samples to 33 ng/μl. 3 μl were used to provide 100ng input. Samples were free of organic solvents and any other contaminations. Controls were prepared with 1 μl of miRNA Assay Controls and 499 μl of RNAse-free water.

The third step, sample annealing, involved an annealing master mix, a combination of 13μl of annealing buffer, 26 μl of nCounter miRNA Tag Reagent and 6.5 μl of the miRNA Assay Control generated in the previous step. Afterward, 3.5 μl of the annealing master mix were combined with 3 μl of total RNA (100ng) prior to starting the annealing protocol in a thermocycler.

The next step was to mix the ligation master mix, 19.5 μl PEG and 13 μl ligation buffer in a microfuge tube, mixing thoroughly by pipetting. 2.5 μl of the ligation master mix per tube were then preheated 5 minutes at 48 °C in a thermocycler and 1 μl of ligase added to each tube.

Prior to hybridisation, 1 μl ligation Clean-Up was added to each tube and topped up with 40 μl of RNAse-free water. Reactions were stored at -20 °C prior to hybridisation.

**miRNA hybridisation protocol**

According to the nCounter® miRNA Expression Assay user manual the final hybridisation reaction contained the following components: 10 µl of the reporter CodeSet, 10 µl hybridisation buffer, a 5 µl aliquot from of the prepared miRNA sample and 5 µl of the capture ProbeSet.

First, aliquots of the reporter CodeSet and capture ProbeSet were mixed and collected with a brief spin. Next, hybridisation buffer (130 μl) was added to the tube containing the reporter CodeSet (130 μl) and 20 μl were pipetted into each tube. MiRNA samples were denatured for 5 minutes at 85°C, chilled quickly on ice and a 5 μl aliquot was added to the hybridisation mix. The thermocycler was programmed for a hybridisation at 65 °C for longer than 12 hours followed by a cool down to 4 °. Before placing the tubes at 65 °C, 5 μl of Capture ProbeSet were to each tube followed by a quick spin. Hybridisation was performed for at least 12 hours and transferred to the nCounter Prep Station.

**Purification and immobilisation**

After hybridisation, samples were transferred to the nCounter Prep Station where excess probes were removed and probe/target complexes were bound, immobilised and aligned on the nCounter Cartridge. Afterwards, the counting and analysis process was initiated on the nCounter digital analyser for data collection.

The raw data was processed using the nSolver software version 4.0. Counts were normalised to eliminate systematic experimental variability, differing amounts of input RNA, along with variability in the background. The seven steps of a basic analysis are explained in the workflow diagram (Figure 3.2).

**nSolver work flow**

In the first step, the data was imported from the nCounter instrument in the form of RCC files using the RCC Import Wizard.

Subsequently, a new experiment was created and the data were normalised using the geometric mean of the counts observed with the positive controls as well as by using the normalising genes in CodeSet. The expression levels for individual miRNAs was then established and the ratio, i.e., fold change was determined
