# Supplementary material for: The Expression and Potential Role of MicroRNAs in Oral Lichen Planus
Source: J Oral Pathol Med. 2026 Feb 3;55(6):642–9. doi: 10.1111/jop.70122 (PMC13333531; doi:10.1111/jop.70122)
Supplement: Supplementary file 2 — Table S1: Demographic and diagnosis of cases (OLP) and controls. [file JOP-55-642-s001.docx]

| **N** | **Age** | **Gender** | **Diagnosis** | **Clinical** | | |  | |  | |
| --- | --- | --- | --- | --- | --- | --- | --- | --- | --- | --- |
| 1 | 60 | M | OLP | Reticular,atrophic | | | | |  | |
| 2 | 75 | F | OLP | Reticular, atrophic | | | | |  | |
| 3 | 38 | M | OLP | Reticular | | |  | |  | |
| 4 | 32 | F | OLP | Reticular, | | |  | |  | |
| 5 | 66 | M | OLP | Reticular, atrophic, erosive | | | | | | |
| 6 | 66 | F | OLP | Reticular, atrophic, plaque-like | | | | | | |
| 7 | 62 | F | OLP | Reticular, atrophic | | | | |  | |
| 8 | 50 | F | OLP | Reticular | | |  | |  | |
| 9 | 45 | M | OLP | Reticular | | |  | |  | |
| 10 | 31 | F | OLP | Reticular, plaque-like | | | | |  | |
| 11 | 63 | M | OLP | Reticular, plaque-like | | | | |  | |
| 12 | 51 | M | OLP | Reticular, plaque-like | | | | |  | |
| 13 | 71 | F | OLP | Reticular | | |  | |  | |
| 14 | 67 | M | OLP | Reticular | | |  | |  | |
| 15 | 71 | M | OLP | Reticular | | |  | |  | |
| 16 | 71 | F | OLP | Reticular | | |  | |  | |
| 17 | 68 | M | OLP | Reticular | | |  | |  | |
| 18 | 45 | F | OLP | Reticular | | |  | |  | |
| 19 | 53 | F | OLP | Reticular/plaque-like | | | | |  | |
| 20 | 53 | M | OLP | Reticular/erosive | | | | |  | |
| 21 | 66 | F | OLP | Reticular | | |  | |  | |
| 22 | 43 | F | OLP | Reticular | | |  | |  | |
| 23 | 62 | M | OLP | Reticular, atrophic | | | | | | |
| 24 | 68 | M | OLP | Plaque-like,reticular | | | | |  | |
|  |  |  |  |  | | |  | |  | |
|  |  |  |  |  | | |  | |  | |
| 1 | 34 | F | Chronic hyperplastic candidosis (candida leukoplakia) | | | | | |  | |
| 2 | 77 | M | Chronic hyperplastic candidosis(candida leukoplakia) | | | | | |  | |
| 3 | 66 | M | Leucoplakia |  | | |  | |  | |
| 4 | 59 | M | Leucoplakia |  | | |  | |  | |
| 5 | 51 | F | Leucoplakia |  | | |  | |  | |
| 6 | 79 | F | Oral hairy leukoplakia | | | |  | |  | |
| 7 | 52 | F | Leukoplakia | |  |  | |  | |  |
| 8 | 33 | F | Leukoplakia with mild dysplasia | |  | | |  | |  |

**Supplementary Table 1-** Demographic and diagnosis of cases (OLP) and controls
